# Hereditary dentine disorders: dentinogenesis imperfecta and dentine dysplasia

**DOI:** 10.1186/1750-1172-3-31

**Published:** 2008-11-20

**Authors:** Martin J Barron, Sinead T McDonnell, Iain MacKie, Michael J Dixon

**Affiliations:** 1 Faculty of Life Sciences and Dental School, Michael Smith Building, University of Manchester, Oxford Road, Manchester, M13 9PT, UK; 2 Dental School, University of Manchester, Oxford Road, Manchester M13 9PT, UK

## Abstract

The hereditary dentine disorders, dentinogenesis imperfecta (DGI) and dentine dysplasia (DD), comprise a group of autosomal dominant genetic conditions characterised by abnormal dentine structure affecting either the primary or both the primary and secondary dentitions. DGI is reported to have an incidence of 1 in 6,000 to 1 in 8,000, whereas that of DD type 1 is 1 in 100,000. Clinically, the teeth are discoloured and show structural defects such as bulbous crowns and small pulp chambers radiographically. The underlying defect of mineralisation often results in shearing of the overlying enamel leaving exposed weakened dentine which is prone to wear.

Currently, three sub-types of DGI and two sub-types of DD are recognised but this categorisation may change when other causative mutations are found. DGI type I is inherited with osteogenesis imperfecta and recent genetic studies have shown that mutations in the genes encoding collagen type 1, *COL1A1 *and *COL1A2*, underlie this condition. All other forms of DGI and DD, except DD-1, appear to result from mutations in the gene encoding dentine sialophosphoprotein (*DSPP*), suggesting that these conditions are allelic.

Diagnosis is based on family history, pedigree construction and detailed clinical examination, while genetic diagnosis may become useful in the future once sufficient disease-causing mutations have been discovered. Differential diagnoses include hypocalcified forms of amelogenesis imperfecta, congenital erythropoietic porphyria, conditions leading to early tooth loss (Kostmann's disease, cyclic neutropenia, Chediak-Hegashi syndrome, histiocytosis X, Papillon-Lefevre syndrome), permanent teeth discolouration due to tetracyclines, Vitamin D-dependent and vitamin D-resistant rickets.

Treatment involves removal of sources of infection or pain, improvement of aesthetics and protection of the posterior teeth from wear. Beginning in infancy, treatment usually continues into adulthood with a number of options including the use of crowns, over-dentures and dental implants depending on the age of the patient and the condition of the dentition. Where diagnosis occurs early in life and treatment follows the outlined recommendations, good aesthetics and function can be obtained.

## Disease name and synonyms

### Dentinogenesis imperfecta (DGI)

Synonyms: Hereditary opalescent dentine; DGI type I; DGI type II; DGI type III; Osteogenesis imperfecta type I with DGI (OI type IA); Osteogenesis imperfecta with opalescent teeth; Brandywine Type DGI; Syndromic DGI, Non-syndromic DGI; DGI without OI, Opalescent teeth without OI; DGI Shields' type II, DGI-I; DGI-II; DGI-III; Capdepont teeth.

### Dentine dysplasia (DD)

Synonyms: Dentine dysplasia type I; Dentine dysplasia type II; DD-I; DD-II; Rootless teeth; Radicular dentine dysplasia.

## Definition and diagnostic criteria

**Figure 1:**
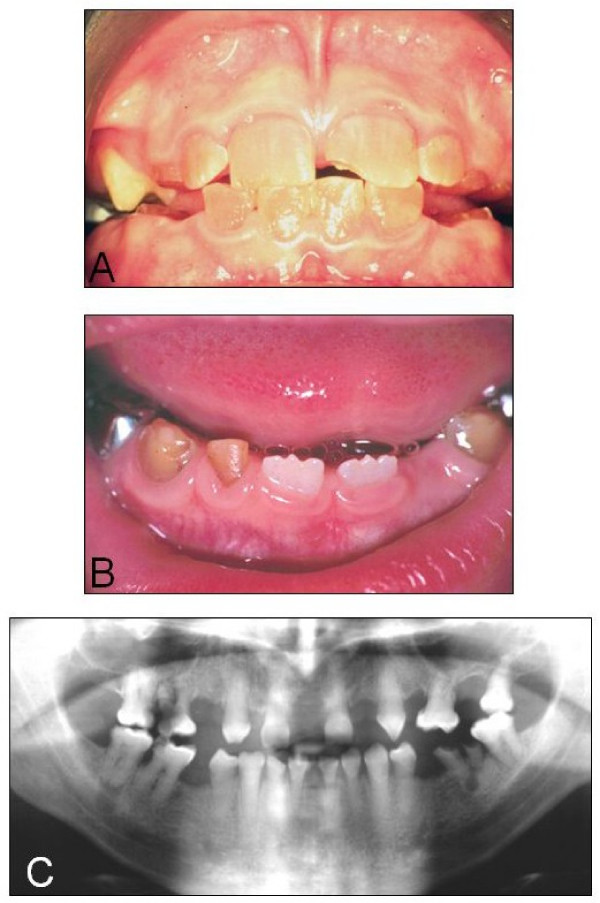
**Clinical features of the inherited dentine disorders**. A. Dentinogenesis imperfecta: The teeth are translucent and often roughened with severe amber discolouration. B. Dentine dysplasia: The primary teeth are translucent and amber in colour whereas the erupting secondary central incisors are of normal appearance. C. Dentine dysplasia: Radiograph showing the thistle-shaped pulp chambers of the secondary teeth.

DGI and DD comprise a group of autosomal dominant genetic conditions characterised by abnormal dentine structure affecting either the primary or both the primary and secondary dentitions. The teeth appear amber, brown/blue or opalescent brown while radiographically the crowns may appear bulbous, pulp chambers are often small or obliterated and the roots are often narrow with small or obliterated root canals [1,2] (Figure 1).

## Epidemiology

Non-syndromic DGI is reported to have an incidence of 1 in 6,000 to 1 in 8,000, whereas the incidence of DD type I is 1 in 100,000 [1,3].

## Classification and clinical description

The classification of hereditary dentine disorders is currently complicated. The most familiar classification system is that formulated by Shields in 1973 [4]. This categorisation discriminates three types of dentinogenesis imperfecta (types I, II and III) and two types of dentine dysplasia (types I and II).

The Shields' system is increasingly out of date as it does not account for the molecular aetiologies of the hereditary dentine defects elucidated so far, for example, those underlying osteogenesis imperfecta and other syndromes manifesting defective dentine formation (see the excellent review by Kim and Simmer 2007 for information on the aetiologies of such syndromes [1]). Unfortunately, the genetic defects that have been discovered to date are insufficient to allow the construction of a comprehensive classification based on our knowledge of the underlying mutations.

The Shields' classification is summarised below:

### Dentinogenesis imperfecta type I

Individuals with DGI-I also have osteogenesis imperfecta. The teeth of both dentitions are typically amber and translucent and show significant attrition. Radiographically, the teeth have short, constricted roots and dentine hypertrophy leading to pulpal obliteration either before or just after eruption. Expressivity is variable even within an individual, with some teeth showing total pulpal obliteration while in others the dentine appears normal.

### Dentinogenesis imperfecta type II

The dental features of DGI-II are similar to those of DGI-I but penetrance is virtually complete and osteogenesis imperfecta is not a feature. Bulbous crowns are a typical feature with marked cervical constriction. Normal teeth are never found in DGI-II. Sensorineural hearing loss has also been reported as a rare feature of the condition [5].

### Dentinogenesis imperfecta type III

This is a form of DGI found in a tri-racial population from Maryland and Washington DC known as the Brandywine isolate. The clinical features are variable and resemble those seen in DGI-I and -II but the primary teeth show multiple pulp exposures and radiographically, they often manifest "shell" teeth *i.e. *teeth which appear hollow due to hypotrophy of the dentine.

### Dentine dysplasia type I

The teeth in DD-I appear generally unremarkable clinically with normal shape, form and consistency. Radiographically, the roots are sharp with conical, apical constrictions. Pre-eruptive pulpal obliteration occurs leading to a crescent-shaped pulpal remnant parallel to the cemento-enamel junction in the permanent dentition and total pulpal obliteration in the deciduous teeth. Numerous periapical radiolucencies are often seen in non-carious teeth.

### Dentine dysplasia type II

The features seen in the deciduous dentition resemble those observed in DGI-II; however, the permanent dentition is either unaffected or shows mild radiographic abnormalities such as thistle-tube deformity of the pulp chamber and frequent pulp stones.

The clinical categories described above do not account for the full range of variation seen in DGI and DD. Thus, bulbous crowns with cervical constriction are not always confined to DGI-II, thistle-tube deformity does not always define DD-II and multiple pulp exposures have been seen in heritable dentine defects other than DGI-III [6,7]. To complicate matters further, the clinical features characteristic of various forms of DGI and DD can be seen in different individuals of the same kindred [8,9]. This variability has led to the suggestion that DD-II, DGI-II and DGI-III are allelic and therefore represent varying degrees of severity of the same disease [10].

In view of the shortcomings of the original Shields' scheme and the lack of sufficient molecular genetic information of the underlying causes of the heritable dentine disorders, a new classification is not yet possible. The most current classification adopted by the Mendelian Inheritance in Man (MIM) database is based on that of Shields [11] but excludes DGI with osteogenesis imperfecta. Thus, the entity once termed DGI-II has now become DGI-I (MIM 125490), while the classification of DGI-III (MIM 125500), DD-I (MIM 125400) and DD-II (MIM 125420) is unchanged. In general, unless otherwise indicated, the Shields' classification, although imprecise, will be followed in this review, as it is currently the more familiar and useful system.

## Aetiology

### Dentinogenesis

Dentine is that component of the tooth which encloses the dental pulp and is itself enclosed, above the gingival margin, by the enamel. Structurally, dentine is composed of a mineral phase of hydroxyapatite (70%), an organic phase (20%) and water (10%) [12]. The organic phase is composed primarily of type I collagen (85%) and the remaining, non-collagenous protein is dominated by dentine phosphoprotein (50%) [12].

Dentinogenesis is a highly ordered process in which the organic predentine matrix is progressively mineralised by ectomesenchymally-derived cells called odontoblasts [12]. The odontoblasts differentiate at the bell stage of tooth development forming a single layer of cells lining the pulp cavity where they secrete the organic predentine matrix into the underlying space [13]. The predentine (10–40 **μ**m thickness) is an unmineralised region containing type I collagen which separates the odontoblast cell bodies from the mineralisation front. At the mineralisation front, the collagenous component of the matrix is thought to provide the correct three-dimensional structure into which the mineral component of dentine is deposited while dentine phosphoprotein, which is secreted from cellular processes extending from the odontoblasts [14], is thought to act as a nucleator of hydroxyapatite crystals during the mineralisation process [12]. As dentinogenesis continues, the odontoblasts continue to migrate deeper into the pulp cavity, extending their processes as they go, while secreting new dentine matrix [12]. The rate of matrix formation exceeds that of mineralisation such that a layer of predentine is always present [12,13]. The first-formed, or mantle, dentine of the tooth crown is approximately 15–20 **μ**m thick and is built upon a dentine matrix containing thick collagen type III fibrils arranged at right angles to the dentine-enamel junction [12]. As the odontoblasts migrate further, the matrix they secrete becomes dominated by finely textured collagen type I fibrils orientated parallel to the dentine-enamel junction, resulting in a denser mineralised dentine known as primary, or circumpulpal, dentine [12]. There are two other types of dentine produced; secondary dentine is formed once root formation has occurred while tertiary dentine forms in response to decay or trauma [12].

### Molecular aetiology

Mutations in the genes encoding the major protein constituents of dentine seem to underlie most hereditary dentine defects.

### Dentinogenesis imperfecta type I

DGI-I is a syndromic form of DGI associated with, and now classified by the MIM database as, osteogenesis imperfecta (MIM 166240). Osteogenesis imperfecta is an autosomal dominant condition usually resulting from mis-sense mutations affecting either of the two genes encoding type I collagen (*COL1A1 *and *COL1A2*) [15]. DGI is occasionally the most penetrant feature in this condition [16].

### Dentinogenesis imperfecta type II, dentinogenesis imperfecta type III and dentine dysplasia type II

DGI-II is now known as DGI-I (MIM 125490) according to the MIM database, whereas DGI-III (MIM 125500), DD-I (MIM 125400) and DD-II (MIM 125420) retain their original classification [4].

The only mutations causative of DGI and DD, with the exception of DD-I for which the underlying mutation(s) has been elucidated, are found in the dentine sialophosphoprotein gene (*DSPP*), suggesting that these conditions are indeed allelic (Table 1). *DSPP *is located within human chromosome 4q22.1 and consists of 5 exons spanning approximately 8343 bp [17]; however, see below for recent information regarding *DSPP *length polymorphisms affecting DPP. *DSPP *is expressed in a number of tissues including bone, kidney, salivary gland and lung but its expression in dentine is hundreds of times higher than in other tissues [5,18-22]. Three distinct protein products are formed from the initially translated polypeptide: dentine sialoprotein (DSP) results from the cleavage of amino acids 16 – 374 of the nascent polypeptide, dentine glycoprotein (DGP) is constituted from amino acids 375 – 462 and dentine phosphoprotein (DPP) is composed of the remaining amino acids of the nascent polypeptide [23-25]. Recent reports suggest that the DPP peptide length varies considerably as the result of in-frame insertion/deletions and single nucleotide length polymorphisms within the highly repetitive, and redundant, DPP portion of the *DSPP *gene [26-28]. Indeed, analysis of DPP from pigs has revealed four distinct porcine alleles that give rise to DPP domains of 551, 575, 589 or 594 amino acids; these length variants are polymorphisms and are not associated with dentine defects [28].

**Table 1:** Mutations of *DSPP *causing DGI and DD.

	***Predicted Protein***	***cDNA***	***Diagnosis***	***Reference***
**Exon 2**	p.Y6D	c.16T>G	DD-II	[31]
	p.A15V	c.44C>T	DGI-II	[33]
	p.P17T	c.49C>A	DGI-II	[23]
	p.P17S	c.49C>T	DGI-II	[2,39]
				
**Intron 2**	p.V18_Q45del	c.52-3C>G	DGI-II	[34]
		c.52-3C>A	DGI-II	[38]
				
**Exon 3**	p.V18F	c.52G>T	DGI-II/III	[5,36-38]
	p.Q45X	c.133C>T	DGI-II	[32,37]
				
**Intron 3**	p.V18_Q45del	c.135+1G>A	DGI-II	[5]
		c.135+1G>T	DGI-II/III	[26]
				
**Exon 5**	p.S624TfsX687	c.1870-1873del TCAG	DD-II	[26]
	p.S640TfsX671	c.1918-1921del TCAG	DD-II	[26]
	p.S680fsX1313	c.2040delC	DD-II	[27]
	p.S758AfsX554	c.2272del A	DGI-II/III	[26]
	p.S842TfsX471	c.2525del G	DGI-II/III	[26]
	p.S865fsX1313	c.2593delA	DGI-II	[27]
	p.S895fsX1313	c.2684delG	DGI-II	[27]
	p.D1146fsX1313	c.3438delC	DGI-II	[27]
	p.D1182fsX1312	c.3546-3550delTAGCAinsG	DGI-II	[27]

Nucleotide numbering follows that adopted in [2], which assumes position 1 is the A of the ATG start codon using the reference sequence NM_014208.3.

DPP is a very repetitive protein that is highly phosphorylated and thought to be involved in the nucleation of hydroxyapatite crystallites and the control of their growth [29]. DPP contains multiple repeats of aspartic acid and phosphoserine mainly as Asp-pSer-pSer and Asp-pSer motifs [29]. Following cleavage, DPP rapidly moves to the mineralisation front where it associates with type I collagen [30]. DSP is a heavily glycosylated protein which forms dimers *via *intermolecular disulphide bridges [30]; however, its function is unknown. DGP contains four phosphorylated serines and one N-glycosylated asparagine [24]. The function of this protein is also currently unknown but it seems likely that it too is involved in the initiation and control of dentine mineralisation.

As a consequence of the repetitive nature of that region of *DSPP *which encodes DPP (exon 5), all of the DGI- and DD-causing mutations that were initially detected were located in the DSP coding region and were composed of mis-sense, non-sense and splicing mutations (Table 1). This observation seems somewhat surprising given that the structure of DSP does not suggest any direct role in mineralisation [2,5,31-39]. Recently, comprehensive analyses of *DSPP *have demonstrated that both DD-II and DGI-II can result from mutations in that region of the gene which encodes DPP. These mutations are exclusively deletions that lead to frame-shifts which change tandem hydrophilic serine-serine-aspartic amino acid repeats to long stretches of hydrophobic residues rich in valine, alanine and isoleucine [26,27]. Moreover, a broad genotype-phenotype correlation has been reported for the DPP mutations with the most 5' mutations, which result in the longest sequences of hydrophobic amino acids, underlying DD-II and the more 3' mutations underlying DGI-II/III; nevertheless, this observation is based on the analysis of a relatively small number of samples and confirmation by screening larger patient cohorts is required [26,27].

Recent information has shown the g.1480A>T [33] variant and g.3595ins18 bp/g.3479del36 bp [35] compound variants [2], to be polymorphisms. The g.1480 A>T mutation has been shown to have allele frequencies of 15% in Finnish [38], 6% in Caucasian [2] and 16% in African-American [2] unaffected control subjects. The compound mutations reported by Dong and co-workers [35], in a family from the Brandywine isolate, were found to resemble similar deletions and insertions in exon 5 present in normal control subjects [2]. Reanalysis of this family by McKnight and co-workers [26], resulted in identification of the mis-sense mutation c.49 C>T, which segregates with the phenotype and causes retention of the protein within the endoplasmic reticulum [2]. Indeed, retention of mutated protein within the endoplasmic reticulum may be a more general mechanism for the molecular pathogenesis of *DSPP*-linked dentine disorders [2,31].

Overall, the mutations in DSPP that have been described to date consist of a combination of mis-sense and non-sense changes, splicing mutations and deletions (Table 1) which have been hypothesised to lead to intracellular retention of DSPP as a consequence of errors in signal peptide or subsequent processing events [2,26,27,31]. Moreover, it has been suggested that all of these mutations will have a dominant-negative effect on the wild-type protein. If this hypothesis is true, it would explain why mice heterozygous for a null allele of *Dspp *appear to have a normal dentition, while their homozygous null littermates exhibit a phenotype similar to that observed in humans affected by DGI-II [40].

Bone defects appear to be absent from individuals with dentine disorders involving mutations of *DSPP *despite DSPP expression in this tissue. This may be due to the low expression level of DSPP in bone [18], altered proteolytic processing [41,42] or molecular redundancy involving other extracellular matrix proteins found in bone [1]. Alternatively, any bone defects may be sufficiently mild to go undetected during clinical examination [1].

Sensorineural hearing loss is associated with the g.49 C>A and g.1197 G>T mutations (MIM 605594) [5]. This is difficult to explain since any hearing impairment found in these patients would be expected to be of the conductive type, involving defects of the ear ossicles, rather than neurosensory. Kim and Simmer [1] suggest the auditory impairment may be an effect secondary to tooth attrition. Overclosure of the jaw is a common consequence of such wearing of the teeth and may lead to altered inner ear shape and concomitant hearing deficits; nevertheless, the cause of the hearing loss remains unresolved.

## Diagnostic methods

### Clinical history

A medical history should aim to establish if the dental condition is a 'syndromic' form of DGI as this is a variable feature of a number of heritable conditions [43] including osteogenesis imperfecta [15], Ehlers Danlos sydrome [44], Goldblatt syndrome [45], Schimke immuno-osseous dysplasia [46], Brachio-skeleto-genital syndrome [47,48], and osteodysplastic and primordial short stature with severe microdontia, opalescent teeth, and rootless molars [49].

Since DGI may be the most penetrant clinical finding in individuals with DGI-I [16], it is very important to ask patients with DGI about histories of bone fracture with minimal trauma, joint hyperextensibility, short stature, hearing loss and scleral hue [2]. A medical history should also aim to establish any conditions the patient has that may aid diagnosis or influence treatment options.

The history should aim to determine that the condition is indeed inherited and not acquired. A family history should establish which other members are affected and allow a pedigree diagram to be compiled. Additionally, a dental history may involve questions which establish whether the primary dentition was also affected and in what way. Details such as colour, tooth wear, abscess formation, tooth mobility and early loss of primary teeth may help to establish what type of DGI or DD the patient has. Dental history, experience and age often influence treatment options and mode of treatment.

### Clinical examination

Obvious extraoral features such as short stature and blue sclera may be consistent with osteogenesis imperfecta. Intraorally, in both dentitions, it is important to consider tooth colour (which may vary from normal to amber, grey or purple to bluish translucent discolouration), tooth wear, abscess formation, tooth mobility and early loss of teeth. The tooth enamel may have sheared off leaving dentine exposed; in such cases the exposed dentine often has a hard glassy appearance due to sclerosis. For this reason, patients rarely complain of sensitivity.

### Radiographs

Radiographs should reveal normal enamel and dentine radiodensity; however, the enamel may already be lost with only dentine remaining. Crowns may appear bulbous with marked cervical constriction. Pulp chambers and canals may be normal, contain pulp stones or, more often, be partially or totally obliterated. Roots are often short but may be of normal length or absent. There may be numerous periapical radiolucencies in non-carious teeth [4].

### Diagnosis

Diagnosis is based on history, clinical examination and radiographic features. DGI-I always occurs in association with osteogenesis imperfecta. The features of DGI-II are similar to DGI-I but osteogenesis imperfecta is not a feature and, because penetrance is almost complete, presentation is more uniform with all teeth affected. Bulbous crowns with marked cervical constriction and pulpal obliteration are a feature of DGI-II and may also present in DD-II. DGI-III is also not associated with osteogenesis imperfecta and, unlike DGI-I and -II and DD-I and -II, it is associated with hypotrophy of dentine and resultant 'shell teeth' which are a distinguishing feature. Like DD-I, DGI-III is associated with multiple periapical radiolucencies in noncarious teeth. DD-I, however, appears normal clinically. Diagnostic radiographic features include sharp conical roots with apical constrictions or rootless teeth, pulpal obliteration with crescent shaped pulpal remnants parallel to the cemento-enamel junction in permanent teeth and total obliteration in primary teeth. The features of DD-II resemble DGI-II in the primary dentition. The permanent dentition, however, is either unaffected or radiographically has thistle tube-shaped deformity of the pulp chamber and pulp stones. Clinical and radiographic features of DGI and DD are summarised in Table 2[50-53]. In reality, the clinical and radiographic presentation is more diverse than the categories described by Shields [4] and classic or diagnostic features of DGI or DD may present in other types [6,7]. In addition, clinical and radiographic features of DGI and DD may differ in individuals of the same family [8,9]. Since pulp chamber and canal obliteration is often progressive, radiographic follow up at various ages is recommended to determine diagnosis [33,54].

**Table 2:** Clinical and radiographic features of dentinogenesis imperfecta and dentine dysplasia [4,9,50-53].

**Dental features**	DGI I	DGI II	DGI III	DD I	DD II
Osteogenesis Imperfecta	+				

Amber translucent (opalescent)	+	+	+/-		+^1^

Attrition	+	+	+/-		+^1^

Bulbous crowns		+	+/-		+^1^

Cervical constriction		+	+/-		+^1^

Pulp exposures			+		

Periapical radiolucencies			+	+	

Shell teeth			+		

Normal roots					+

Short constricted roots	+	+	+/-		+^1^

Sharp conical short roots				+	

Rootless teeth				+	

Pulp obliteration primary	+/-	+		+	+

Pulp obliteration permanent	+/-	+			

Partially obliterated crescent shaped pulp chamber Permanent				+	

Thistle tubed pulp chamber					+/-^2^

Pulp stones				+/-	+/-^2^

Primary Dentition affected	+	+	+	+	+

Permanent dentition affected	+	+	+	+	+/-

No clinical features				+	+^2^

Variable expression	+		+		+

^1 ^Primary Dentition.^2 ^Permanent dentition.

## Differential diagnosis

Included in the differential diagnosis are conditions that have similar clinical or radiographic features to DGI or DD.

### I. Clinical

#### a) Exposure of underlying dentine

Hypocalcified forms of amelogenesis imperfecta initially develop normal enamel thickness but the poorly calcified enamel is soft and friable and is rapidly lost by attrition leaving dentine cores. Unlike DGI the teeth are usually sensitive and on radiographs enamel is less radio-dense than dentine [9]. Pulp chamber and root canals are usually not sclerosed.

#### b) Intrinsic discolouration

Congenital erythropoietic porphyria is a rare condition resulting from an inborn error of porphyrin metabolism. This deficiency leads to haemolytic anaemia, photosensitivity, blistering of the skin, and deposition of red-brown pigments in the bones and teeth [55]. A number of prenatal and neonatal enamel discolourations and hypoplasias are due to neonatal haemolytic anaemias. Most cases are due to Rhesus incompatibility. The discolouration which ranges from yellow through to green, brown and grey to black is usually found at the necks of teeth and the enamel hypoplasias are usually located in the coronal third of the teeth [56].

Tetracyclines have the ability to chelate calcium ions and to be incorporated into developing teeth, cartilage and bone, resulting in discolouration of both the primary and permanent dentitions. This permanent discolouration varies from yellow or grey to brown depending on the dose or the type of the drug received in relation to body weight [57].

#### c) Mobility leading to early tooth loss

Other causes of early loss of teeth as in DGI-III and DD-I include: hypophosphatasia, immunological deficiencies *e.g. *severe congenital neutropenia (Kostmann's disease), cyclic neutropenia, Chediak-Hegashi syndrome, neutropenias, histiocytosis X, Papillon-Lefevre syndrome and leucocyte adhesion deficiency syndrome [58]. With the exception of hypophosphatasia, all of these conditions have an underlying immunological defect which makes those with these conditions susceptible to periodontal breakdown. Mobility of teeth in those with hypophosphatasia however is due to aplasia or marked hypoplasia of cementum.

### II. Radiographic

Regional odontodysplasia is of unknown aetiology. Radiographically, roots are short with wide open apices and very wide pulp canals, and often become infected as in DGI-III and DD-I. Primary and permanent teeth are affected [59]. Irradiation to jaws or chemotherapy during the period of root development leads to arrested development and can give a radiographic appearance of DD-I [60].

### III. Clinical and radiographic

Vitamin D-dependent rickets and vitamin D-resistant rickets have clinical and radiographic features of DGI and DD. Vitamin D-dependent rickets is characterised by yellowish to brown enamel, chronic periodontal disease, large quadrangular pulp chambers and short roots [61]. Features of vitamin D-resistant rickets include attrition and exposure of abnormally formed dentine of primary teeth and abscessed non-carious primary or permanent teeth [62].

As previously mentioned, DGI-I is a variable feature of Ehlers Danlos syndrome [44], Goldblatt syndrome [45], Schimke immuno-osseous dysplasia [46] and Brachio-skeleto-genital syndrome [47,48], and osteodysplastic and primordial short stature with severe microdontia, opalescent teeth, and rootless molars [49].

## Genetic counselling

As DGI and DD are inherited in an autosomal dominant fashion, there is a 50% chance that a child born to an affected parent will themselves be affected.

In general, the diagnosis is made on clinical grounds alone; however, as the genetic mutations underlying these conditions are delineated, molecular genetic diagnosis may prove to be a useful adjunct to clinical analysis, particularly where the precise diagnosis is in doubt.

## Management

The aims of treatment are to remove sources of infection or pain, restore aesthetics and protect posterior teeth from wear. Treatment varies according to the age of the patient, severity of the problem and the presenting complaint.

In the primary dentition, stainless steel crowns on the molars may be used to prevent tooth wear and maintain the occlusal vertical dimension. The aesthetics may be improved using composite facings or composite strip crowns [63,64]. If, however the child presents late, the teeth may have undergone attrition to the level of the gingivae and the only treatment option then is to provide over-dentures [65,66]. Children usually adapt well to over-dentures but they need to be reviewed regularly and dentures remade, as the child grows. If abscesses develop, pulp therapy is not successful and removal of the affected teeth is required. In younger children, where co-operation is limited, or the level of treatment required is extensive, a general anaesthetic may be required to facilitate treatment. In some cases, the parents or child may not be concerned with aesthetics and may request removal of sources of pain or infection only.

As the permanent dentition erupts, it should be closely monitored in relation to the rate of tooth wear with intervention only if necessary. Cast occlusal onlays on the first permanent molars and eventually the premolars, help to minimise tooth wear and maintain the occlusal vertical dimension [67]. The emphasis should be on minimal tooth preparation until the child reaches adulthood. At this point, if clinically indicated, a full mouth rehabilitation may be considered. Teeth with short thin roots and marked cervical constrictions however are often unfavourable for crowns [67]. Obliteration of the pulp chambers and root canals in teeth that develop abscesses makes endodontic therapy difficult if not impossible. Successful conventional endodontic therapy however has recently been reported in a case with DD-I [68]. If conventional therapy is not an option, periapical curettage and retrograde root filling is another possible alternative, but is not recommended for teeth with short roots [69].

Some patients present with severe tooth wear in the permanent dentition. As in the primary dentition, one of the options is over-dentures. Those with DD-I have mobile teeth due to very short roots and as a result tend to lose teeth early in the primary and permanent dentition. Until growth is complete, the treatment of choice for the replacement of missing teeth is dentures. Dental implants may be considered when growth is complete at about 18 years of age. Maxillo-mandibular atrophy is a consequence of no or rudimentary root development and early tooth loss. Ridge augmentation prior to implants is often required [70,71].

Exposed dentine is more susceptible to tooth decay than enamel. For all patients, regular dental checkups and prevention of tooth decay in the form of oral hygiene instruction, dietary advice and appropriate use of fluoride is essential. Early diagnosis and regular dental care however cannot prevent premature tooth-loss due to short or absent roots and spontaneous abscess formation that occurs in some types of DGI and DD [72].

## Prognosis

The outcome of a diagnosis of DGI/DD largely depends upon the age at which the diagnosis was given and the speed and quality of the treatment provided. Where diagnosis occurs early in the life of the patient and treatment follows the recommendations outlined above, good aesthetics and function can be obtained thereby minimising nutritional deficits and psychosocial distress.

## Abbreviations

DGI: Dentinogenesis imperfecta; OI type IA: Osteogenesis imperfecta type I with DGI; DD: Dentine dysplasia; MIM: Mendelian Inheritance in Man; DSP: dentine sialoprotein; DGP: dentine glycoprotein; DPP: dentine phosphoprotein.

## Competing interests

The authors declare that they have no competing interests.

## Authors' contributions

The authors contributed to this review article. They read and approved the final version of the manuscript.
